# 4020 Impact of Patients’ Health Literacy Level on Patients’ Health Outcomes

**DOI:** 10.1017/cts.2020.406

**Published:** 2020-07-29

**Authors:** Dae Hyun Kim, Larry Hearld, William Opoku-Aygeman

**Affiliations:** 1 University of Alabama at Birmingham

## Abstract

OBJECTIVES/GOALS: The objective of this study is to examine the relationship between gastro-intestinal (GI) patients’ health literacy levels and patients’ health outcomes (length of stay, readmission, complication). METHODS/STUDY POPULATION: A research team at the University of Alabama at Birmingham (UAB) ‘s Gastro-Intestinal (GI) surgical department collected inpatient GI patients’ health literacy data by distributing the Brief Health Literacy Screen (BRIEF) survey to patients are about to be discharged. Patients’ health outcomes data were gathered through Business Objects, an online platform that allows physicians and researchers to access and gather patients’ medical information with an IRB approval. After accounting for necessary control variables, logistic regression and multiple linear regression models will be run to assess whether there is a significant relationship between patients’ health literacy levels and patients’ health outcomes. RESULTS/ANTICIPATED RESULTS: Three specific hypotheses are proposed in this study. H1: GI patients’ health literacy levels will be negatively associated with their lengths of stay H2: GI patients’ health literacy levels will be negatively associated with their readmission status to the hospital H3: GI patients’ health literacy levels will be negatively associated with their complication status to the hospital DISCUSSION/SIGNIFICANCE OF IMPACT: This study allows us to further our understanding of patients’ health literacy level and its’ relationship with important health outcomes. By looking at a variety of diverse health outcomes, the impact of a patients’ health literacy level on that patients’ health outcomes will be observed more clearly.

